# Correlation between initial tumour volume and treatment duration on Dabrafenib: observation study of subjects with BRAF mutant melanoma on the BRF112680 trial

**DOI:** 10.1186/s12885-020-06848-8

**Published:** 2020-04-22

**Authors:** Arwa Ali, Monica Dumbrava, Kylie Riddell, Nina Stewart, Robyn Ward, Ahmed K. Ibrahim, Melvin Chin

**Affiliations:** 1 Medical Oncology, Nelune Comprehensive Cancer Centre/The Bright Alliance Building, Prince Of Wales Hospital, Randwick, NSW 2031 Australia; 2 Medical Oncology Department, South Egypt Cancer Institute, Assiut University, Asyut, Egypt; 3 Medical Oncology Department, North West Regional Hospital, Burnie, Tasmania Australia; 4 GlaxoSmithKline Research and Development, Ermington, Australia; 5 Radiation Oncology Department, Fiona Stanley Hospital, Murdoch, WA Australia; 6 Faculty of Medicine and Health, The University of Sydney, Camperdown, NSW Australia; 7 Community Health School, Faculty of Medicine, Assiut University, Asyut, Egypt

**Keywords:** Tumor volume, RECIST-base assessment, Predictive biomarkers, BRAF-inhibitors, Treatment duration, Metastatic melanoma

## Abstract

**Background:**

Planar-based measurements of lesions in metastatic melanoma have limitations in estimating tumor burden of a patient and in predicting response to treatment. Volumetric imaging might add predictive value to Response criteria in Solid Tumor (RECIST)-measurement. Based on clinical observations, we explored the association between baseline tumor volume (TV) and duration of treatment with dabrafenib in patients with metastatic melanoma. We have also explored the prognostic value of TV for overall survival (OS) and progression free survival (PFS).

**Methods:**

This is a retrospective, chart-review of primary source documents and medical imaging of a cohort of patients participating in the BRF112680 phase 1 clinical trial at the Prince of Wales Hospital. TV was quantified by contouring all the measurable baseline target lesions in the standard manner for radiation planning using Voxxar™ software. We used Cox regression models to analyse associations between TV and duration of treatment with dabrafenib and between TV, PFS and OS.

**Results:**

Among 13 patients of BRAF 112680 trial, 10 were included in the retrospective analysis. Target lesion sum volume ranged from 0.3 to 1065.5 cm^3^ (cc), with a median of 27.5 cc. The median PFS and OS were 420 days (range 109–1765) and 1680 days (range 390–2940), respectively. The initial TV was inversely correlated with duration of treatment with dabrafenib (rho − 0.6; *P* 0.03). In multivariate analysis, TV was a predictor for OS (HR 2.81 CI 1.06–6.19) and PFS (8.76 (CI 1.05–43.58). Patients with tumour volume above the median had significantly lower OS of 6-months compared to 56-months survival for patients with smaller volumes; *P =* 0.019.

**Conclusions:**

TV is a predictor for treatment duration and is prognostic of OS and PFS in patients with metastatic melanoma. These findings need to be validated prospectively in clinical trials.

## Background

The BRAF inhibitors have significantly changed the outcomes for patients with BRAF mutant metastatic melanoma by improving survival and reducing symptoms by inducing tumour response, when compared with chemotherapy [1–5].

In early and intermediate stage melanoma, it has been shown that tumour volume (TV) estimated by Cavalieri’s method, is a practical and reproducible variable, which quantifies tumour burden in accordance with tumour biology and has demonstrated predictive value in multivariate analyses [6–9] [10]. However, in metastatic melanoma (MM), the unidimensional response evaluation criteria in solid tumors (RECIST) and bi-dimensional variables or WHO measurements (which are based on clinical examination) are the most commonly used surrogates of TV for assessing response. Recently, studies have shown that CT tumor volume measurements using segmentation tools have been consistently more reproducible than diameter measurements used in RECIST [11], and reflect the entire TV rather than planar measurements on one axial plane image [12]. Yet, using TV parameter is still evolving and need to be prospectively validated in the clinical trials.

In 2009, the BRF112680 phase 1 clinical trial -first in human- investigating the safety and tolerability of dabrafenib was opened at our institution (Prince Of Wales Hospital, NSW). A total of 13 patients with metastatic cancers, mainly melanoma, were recruited. Three patients who had MM remained on dabrafenib monotherapy for more than 4 years, compared with the median PFS of 5–6 months in reported studies. An observation was made that these long-term survivors had qualitatively lower volumes of disease at presentation than other patients. This study aimed to explore if this clinical observation does indeed represent a signal of initial tumour volume being a predictive factor for response to treatment. Our primary objective was to study the association between measured total initial TV and the duration of treatment on DF. The secondary objectives were its association with overall survival (OS), progression free survival (PFS).

## Methods

### Study design

This is a retrospective, chart review of primary source documents and medical imaging of a cohort of patients participated in the BRF112680 phase-1 clinical trial at the Glaxo Smith Kline (GSK) Medicines Research Unit at Prince of Wales Hospital. The patients with BRAF-V600E mutation positive, unresectable or metastatic melanoma were selected. Total initial TV was defined as the sum of measured volumes of all metastatic lesions at all metastatic sites seen on the baseline pre-enrolment CT scan of that individual. Duration of treatment was defined as the interval between commencement and permanent cessation of dabrafenib on trial. PFS was the interval between first dose of dabrafenib and date of disease progression or death due to any cause. Progression was defined as ≥20% increase in the smallest sum of study or new lesions of new lesions as per RECIST 1.1 criteria. Overall survival was defined as the interval between the first dose of dabrafenib and death due to any cause, end of study period or lost to follow up. The phase 1 study, BRF112680- A Phase I, Open-Label, Multiple-Dose, Dose-Escalation Study to Investigate the Safety, Pharmacokinetics, and Pharmacodynamics of the BRAF Inhibitor GSK2118436 in Subjects with Solid Tumors was approved by the Bellberry Human Research Ethics Committee (B65/09) and signed informed consent were obtained from all patients that included the publication of the study results in medical journals. The protocol of this further analysis of the role tumour volume and treatment duration was separately assessed and approved by the South Eastern Sydney Local Health District Human Research Ethics Committee (LNR/14/POWH/539) which found that this study involved no further disclosure than which was expected by the patients when consenting to the original study.

### Study population

This retrospective study was performed on 13 patients with BRAF-mutant metastatic cancer enrolled in one centre of the BRF112680 trial. The phase I trial commencing in 2009 was conducted with an accelerated dose titration design. This centre enrolled the first few patients in this first-in-man trial of dabrafenib. Three patients had colorectal cancer and had been excluded from the study. The inclusion criteria (as per the BRF 112680 study) were; histologically confirmed solid tumour for which no curative treatment was available, 18 years or older, Eastern Cooperative Oncology Group (ECOG) performance status of 1 or less (patients with an ECOG status of 2 could be enrolled with the approval of the study’s medical monitor), life expectancy of 3 months or longer, absence of known progressing or unstable brain metastases, adequate hematologic, hepatic, and renal function. The presence of a BRAFV600E mutation was mandatory. The exclusion criteria were; age < 18 years, patients with BRAF wild-type melanoma or with unknown BRAF mutation status, patients previously treated with BRAF inhibitors, patients with moderate or severe hepatic impairment. Upon successful escalation of dose cohort, patients on lower dose levels were given the increased dose, which for the patients in this trial, was subsequently 100 mg TID or 150 mg BD [13].

### Management of data

Patients enrolled in BRF112680 trial were assigned a study number. However, as all patients had baseline CT scans in the hospital, they were also given a hospital medical record number through which archived medical images were stored. Tumour volume measurements were done on patient’s identified scans but were calculated by the investigator who was not involved in the BRF 112680 phase 1 trial, to reduce observer bias, and entered into the TV worksheet. Data extracted from the source documents was collected by investigators involved in the phase I trial using the BRF112680 study number. Once primary data collection was completed and verified, the work sheets containing the patient’s identifiers had the primary identifiers erased, leaving only the study number. Any further analysis was done on the de-identified but re-identifiable data. The total initial tumour volume was measured using Focal 4D™ software. The baseline staging CT for each patient was loaded from the hospital PACS™ to Voxxar™ software and imported into a workstation containing the Focal 4D™ software. By using the formal radiological and PET reports, we identified the organs involved with metastatic disease for each patient. The sites and the number of organs involved in each patient (eg. liver, lung, lymph nodes, soft tissues, bones, brain) were also recorded.

Tumour volume was calculated by contouring the lesions in the standard manner as used by Radiation Oncologists to outline the gross tumour volume for radiation planning:

- Drawing a contour for each lesion, using the same colour label for any lesions seen in the same organ. After contouring all lesions in an organ, the software will automatically calculate a tumour volume of that organ. The number of metastases in each organ will also be recorded automatically.
- The sum of the tumour volumes in all organs identified to have metastatic disease provided the overall initial tumour volume for the patient.

### Statistical methods

Data were verified, coded by the researcher and analysed using IBM-SPSS™ 21.0 (IBM-SPSS Inc., Chicago, IL, USA) [14]. Descriptive statistics: means, standard deviations, medians, IQR and percentages were calculated. Predictors of the TV were tested using multivariable linear regression analysis (likelihood Ratio Test). The prognostic effect of the various parameters on clinical outcome was tested using the Kaplan–Meier method with the log-rank test was applied to compare survival curves. Multivariate analysis was carried using the Cox regression model for OS and PFS. A significant *p* value was considered when it is equal or less than 0.05.

## Results

### Patients’ characteristics

From the total of 13 patients enrolled in the BRF 112680 phase I trial at our site, three patients were excluded from this analysis as they had non-melanoma diagnoses. The final series comprised 10 patients, eight men and two women (median age 61.5 years, range 28–81 years). The main clinical, biological, and radiological characteristics of patients are shown in Tables 1 and 2. Collection of serum lactate dehydrogenase (LDH) was not mandated in the clinical trial. Results were available for eight patients, six (75%) of them has level above the normal upper limit. Comorbidities were documented in six patients. None of the patients had brain metastases on trial commencement.

**Table 1:** Socio-demographic and Clinical characteristics of the studied Cohort

Variable	Category	***n*** = 10
**Age Group**	Mean ± SD	57.63 ± 16.2
	Median (IQR)	61.5 (20)
**Sex**	Male	8 (80%)
	Female	2 (20%)
**ECOG at Baseline**	0	4 (40%)
	1	6 (60%)
Co-morbidity*	Positive	6 (60%)
	Negative	4 (40%)
**No. Involved Organs**	1	4 (40%)
	> 1	6 (60%)
**TV (cc)**	Mean ± SD	111.50 ± 59.6
	Median (IQR)	27.5 (80)
**LDH (120–250 Unit/litre)**	Mean ± SD	290.38 ± 92.8
	Median (IQR)	258.5 (114)
**RECIST Response**	PR	**3 (30%)**
	Stable	**2 (20%)**
	PD	**5 (50%)**
**Survival data**	Dead	3 (30%)
	Alive	7 (70%)

* Co-morbidities included diabetes mellitus (DM), hypertension, ischemic/valvular heart disease, glaucoma, benign prostatic hyperplasia

*ECOG* European Cooperative Oncology Group, *IQR* interquartile range, *LDH* lactate dehydrogenase, *PR* partial remission, *PD* progressive disease, *RECIST* response criteria in solid tumors, *SD* standard deviation, *TV* tumor volume

**Table 2:** Summary of study cohort clinical characteristics

Case No	age	LDH	DF Duration	No organs	Organs involved	TV (cc)	RECIST
1	36	NA	1512	1	Lymph nodes (LN)	1.93	SD
2	41	NA	237	5	LN, lung, soft tissues, bowel, liver	1065.58	SD
3	58	266	252	3	Liver, bone, lung	40.39	PR
4	66	226	1765	1	lung	0.3	PR
5	67	363	109	2	LN, liver	74.2	PD
6	28	193	126	2	LN,liver	14.06	PD
7	46	484	125	3	Lung, liver, LN	445.4	PD
6	65	251	637	1	lung	5.61	PR
5	50	292	1586	2	Lung, liver	7.62	PD
10	81	548	956	3	s/c tissues, thoracic LN, intra-abdominal deposits	304.48	PD

*ECOG* European Cooperative Oncology Group, *IQR* interquartile range, *LDH* lactate dehydrogenase, *LN* lymph nodes, *PR* partial remission, *PD* progressive disease, *RECIST* response criteria in solid tumors, *S/C* subcutaneous, *SD* standard deviation, *TV* tumor volume

Target lesion sum volume ranged from 0.3 to 1065.5 cm^3^ (cc), with a median of 27.5 cc. Two patients had only one organ involved. Organs of involvement are depicted in **(**Table 2**).**

**Fig. 1:**
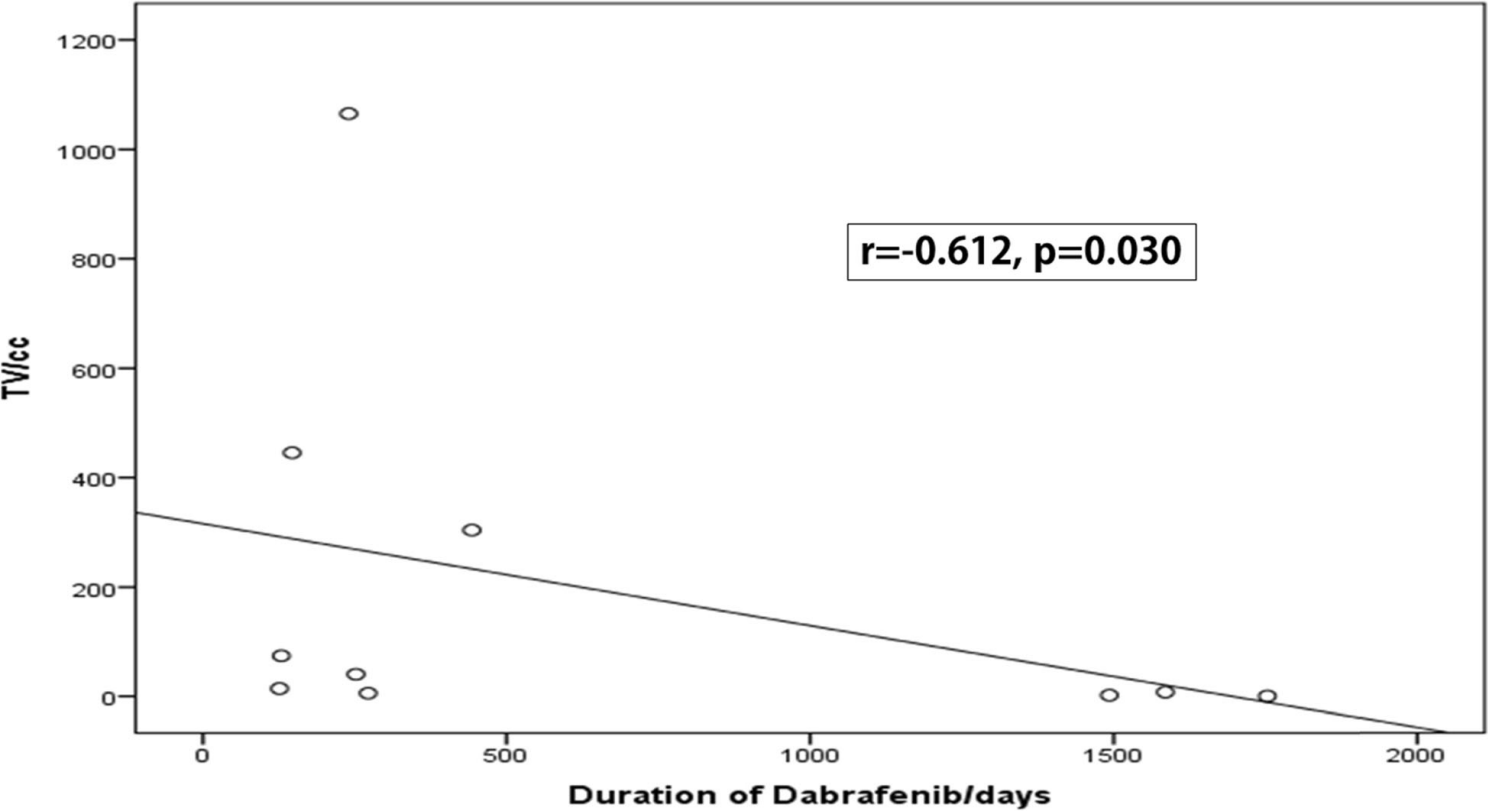
Correlation between Tumour volume and Duration of Dabrafenib Treatment. Rho = Spearman’s correlation coefficient, TV = tumor volume

**Fig. 2:**
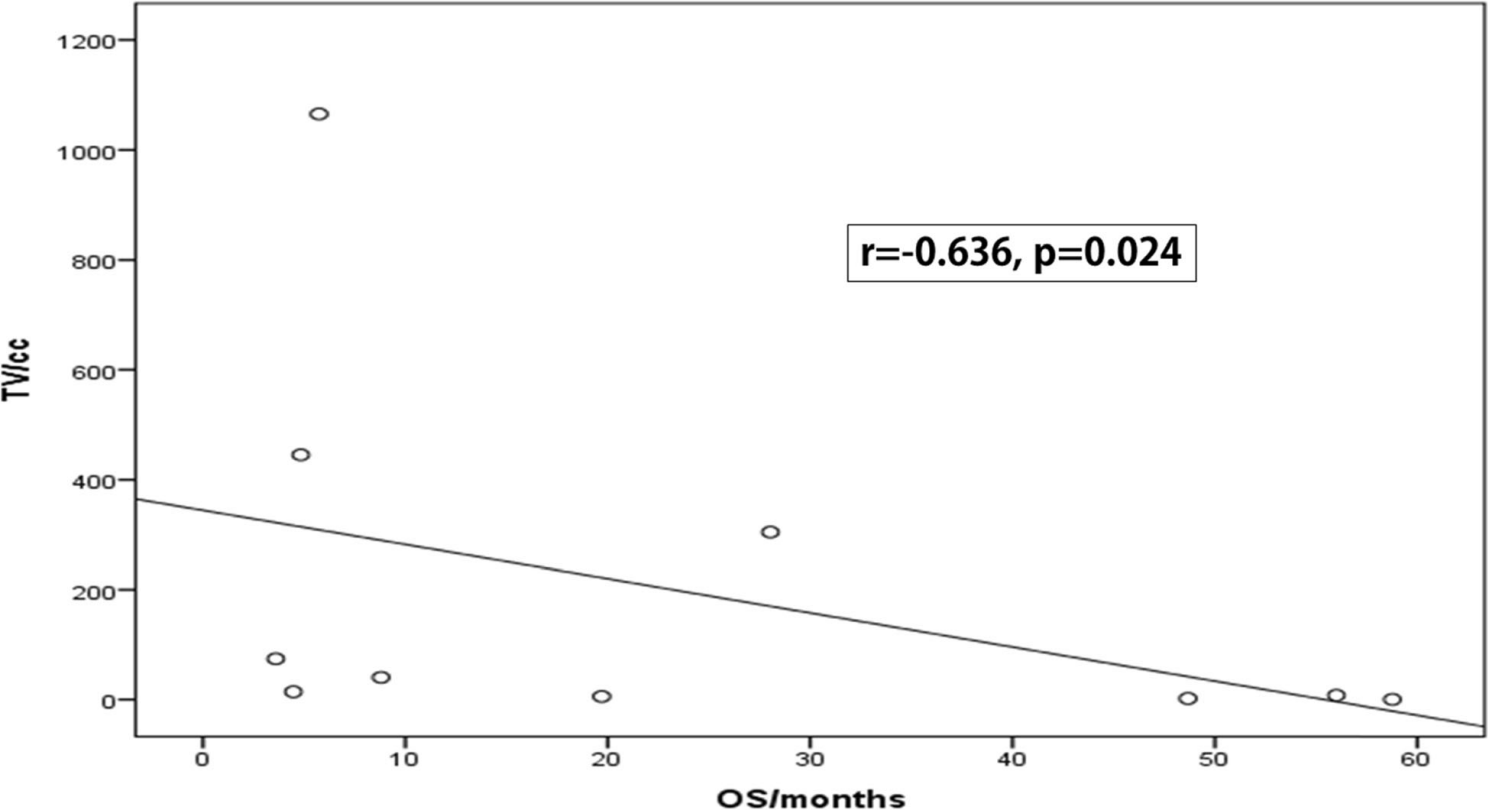
Correlation between Tumour volume and OS time. OS = overall survival, Rho = Spearman’s correlation coefficient, TV = tumor volume

There was an inverse correlation between the initial TV and the duration of dabrafenib treatment; rho was − 0.612 *p* = 0.03 **(**Fig. 1**)**. Likewise, there was negative correlation between the initial TV and the overall survival time; rho was − 0.636 *p* = 0.024 **(**Fig. 2**)**.

The median PFS and OS were 420 days (range 109–1765) and 1680 days (range 390–2940), respectively. Death occurred in 30% of patients during the clinical trial period (3/10). On trial, half the patient attained partial response and stable disease according to RECIST criteria and disease progression was the outcome in the other half.

Regarding the OS predictors; the multivariate analysis and after adjusting for age, the final cox-hazard regression model contained two predictors; TV and RECIST response. For the TV, with 1 cc increase in TV there was three folds increment in the mortality hazard; HR was 2.81 (CI 1.06–6.19) and this was statistically significant (*p* = 0.047). Moreover, patients with progression of disease (PD) were three times more likely to die compared with those with either stable or regressive conditions; HR was 3.24 (CI 1.01–22.38), *p* = 0.044 **(**Table 3**).**

**Table 3:** Cox Proportional Hazard Regression analysis for OS

Variable	Univariate analysis		Multivariate analysis	
	HR (95% CI)	***P***-value	HR (95% CI)	***P***-value
**Age/years**	0.998 (0.917–1.087)	= 0.971	1.144 (0.681–1.922)	**= 0.610**
**ECOG**	0.707 (0.042–11.79)	= 0.809		
**TV (cc)**	1.005 (0.983–1.029)	= 0.639	2.814 (1.059–6.189)	**= 0.047**
**LDH**	0.909 (0.631–1.311)	= 0.610		
**RECIST response (PD)**	4.243 (0.255–17.15)	= 0.314	3.242 (1.005–22.38)	**= 0.044**

*CI* confidence interval, *ECOG* European Cooperative Oncology Group, *HR* hazard ratio, *LDH* lactate dehydrogenase, *PR* partial remission, *PD* progressive disease, *RECIST* response criteria in solid tumors, *SD* standard deviation, *TV* tumor volume

In the PFS multivariate analysis, after adjusting for age and sex, the TV showed a signal as a potential predictor of PFS; HR was 8.76 (CI 1.05–43.58) *p* = 0.048. Also, the serum LDH level presented a signal as a potential predictor of PFS; HR was 1.05 (CI 1.004–4.27) *p* = 0.047.

**Fig. 3:**
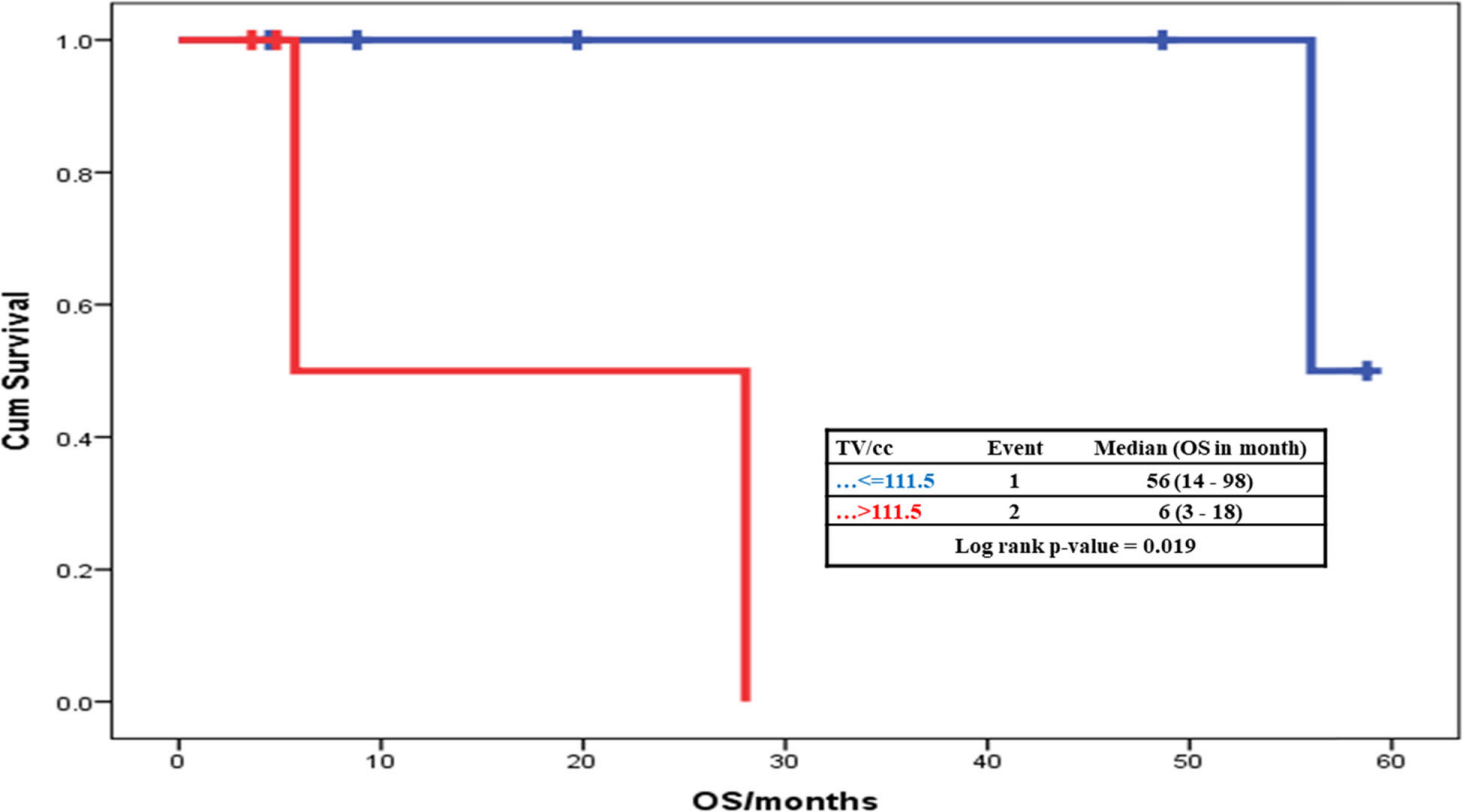
Effect of TV on the OS of the Studied Cases. OS = overall survival, TV = tumor volume

When dichotomized by the TV median, patients with volume above the median had significantly lower OS of 6-months compared to 56-months survival for patients with smaller volumes; *P =* 0.019 **(**Fig. 3**)**. Similarly, patients with volume above the median had significantly lower PFS of 4-months compared to patients with smaller volumes who lived longer than the median PFS; *P =* 0.013.

## Discussion

The existing prognostic variables for advanced melanoma, such as the number and sites of metastatic disease [15, 16] are semi-quantitative. Quantitative imaging, such as volumetric CT, may represent a useful in-vivo biomarker, allowing adaptive trial designs and facilitating management decisions in clinical settings.

Our study has confirmed TV as an independent predictive factor for duration of dabrafenib treatment. To our knowledge, there are no previous studies which have rigorously examined the correlation between TV and duration of anti-BRAF treatment as a surrogate for clinical benefit. In addition, this case series provides a signal for TV as having prognostic value for PFS and OS, with both *P* values of 0.04. Some recent studies have reported the impact of baseline tumor burden as assessed by CT scans on survival. For example, in the KEYNOTE-1 study, the sum of the maximum diameters of target lesions on the baseline scan (baseline tumor size) was used as an index of tumor burden. A higher burden was associated with significantly worse OS [17]. In another study, that semi-automated analysis of MRI-tumor volume (initial and final change) was a better predictor of recurrence free survival than the longest dimension measured manually by RECIST or WHO guidelines in breast cancer patients treated with neoadjuvant chemotherapy [18] [19].

There are limitations to using tumor volumes and more broadly all anthropomorphic measurements as predictive biomarkers. For example, it was noted that diameter-based RECIST measurements may not be reflective of the actual changes in the tumor mass or being reflective of the appearance of new lesion within complex masses. Hence, static tumor volume is not an adequately useful biomarker [20]. Hitherto, the dynamics of TV growth rate rather than a static volume measurement has been proposed as an emerging tool to predict disease response in many solid tumours including lung cancer [21] [22] [23, 24], head and neck tumor [12], and adult high grade gliomas [25, 26]. For example, in a cohort of EGFR-mutant advanced non-small-cell lung cancer (NSCLC) patients treated with erlotinib or gefitinib, the 8-week tumor growth rate has been shown to correlate with survival. A greater tumor volume decrease at 8 weeks was associated with longer OS (*p* = 0.01) [23]. Similarly, in another series of 42 ALK-mutant lung cancer treated with crizotinib, the higher 8-weeks volume reduction was significantly associated with longer survival [24]. Taken together, there is a mounting evidence suggesting that volumetric image analysis adds value to clinical trial science in terms of sensitivities and precision particularly in geometric challenges such as spiculated masses, infiltrative tumors that lack clear margins or tumours of small size [27].

## Conclusions

Although our study is limited by the small number of patients analysed and the retrospective design, but it has confirmed the observation of a significant inverse relationship between tumor volume and clinical benefit with treatment on dabrafenib. If prospectively validated, initial tumour volume may represent a predictive factor to consider when evaluating results of oral kinase inhibitors and a prognostic factor for clinicians regarding duration of clinical benefit.

## Data Availability

All data generated or analysed during this study are included in this published article.

## Abbreviations

ALK: Anaplastic lymphoma kinase
BD: Twice daily “bis in die”
CC: Cubic centimetres
CI: Confidence interval
CT: Computed tomography
ECOG: Eastern Cooperative Oncology Group
EGFR: Epidermal growth factor
GSK: Glaxo Smith Kline
HR: Hazard ratio
HREC: Human Research Ethics Committee
IQR: Inter quartile range of observation
LDH: Lactate dehydrogenase
MM: Metastatic melanoma
MRI: Magnetic resonance imaging
NSCLC: Non-small cell lung cancer
NSW: New South Wales
OS: Overall survival
PFS: Progression free survival
RHO: Coefficient of linear correlation
RECIST: Response criteria in solid tumor.
SESLHD: South Eastern Sydney Local Health District
TID: Three times daily “ter in die”
TV: Tumor volume
WHO: World Health Organisation
